# Chilling-Dependent Release of Seed and Bud Dormancy in Peach Associates to Common Changes in Gene Expression

**DOI:** 10.1371/journal.pone.0035777

**Published:** 2012-05-10

**Authors:** Carmen Leida, Ana Conejero, Vicent Arbona, Aurelio Gómez-Cadenas, Gerardo Llácer, María Luisa Badenes, Gabino Ríos

**Affiliations:** 1 Instituto Valenciano de Investigaciones Agrarias (IVIA), Valencia, Spain; 2 Departamento de Ciencias Agrarias y del Medio Natural, Universidad Jaume I, Castellón de la Plana, Spain; United States Department of Agriculture, United States of America

## Abstract

Reproductive meristems and embryos display dormancy mechanisms in specialized structures named respectively buds and seeds that arrest the growth of perennial plants until environmental conditions are optimal for survival. Dormancy shows common physiological features in buds and seeds. A genotype-specific period of chilling is usually required to release dormancy by molecular mechanisms that are still poorly understood. In order to find common transcriptional pathways associated to dormancy release, we analyzed the chilling-dependent expression in embryos of certain genes that were previously found related to dormancy in flower buds of peach. We propose the presence of short and long-term dormancy events affecting respectively the germination rate and seedling development by independent mechanisms. Short periods of chilling seem to improve germination in an abscisic acid-dependent manner, whereas the positive effect of longer cold treatments on physiological dwarfing coincides with the accumulation of phenylpropanoids in the seed.

## Introduction

Perennial plants protect important and delicate tissues as reproductive meristems and embryos in specialized structures respectively designated buds and seeds. Growth of bud and embryo meristems leading respectively to blooming and germination is strictly regulated by dormancy mechanisms, which impose a physiological constraint to this growth until environmental conditions are optimal for long-term survival [1]–[5]. Dormancy is released in different species by post-harvest storage of seeds (after-ripening), moist chilling of seeds (stratification) or a prolonged period of chilling in buds. In stone-fruit species, a high correlation between the chilling requirements for seed and bud dormancy release has been observed [6], [7], which suggests the presence of common regulatory mechanisms. Insufficient cold stratification of seeds in peach (*Prunus persica*), almond (*Prunus dulcis*), and other rosaceous plants may cause, in addition to low germination rates, a shoot development abnormality called physiological dwarfing [8]. Physiological dwarfs are characterized by a temperature-dependent rosette-type habit of growth, with short internodes, and deformed leaves [9].

Seed dormancy has been observed throughout higher plants with physiological and morphological particularities in different species [10]. In most popular model organisms, physiological dormancy integrates contributions from the embryo and the seed coat, being the coat component at least partially due to the mechanical resistance to breakage of endosperm and testa layers [11]. The ratio of the hormones abscisic acid (ABA) and gibberellins (GA) is considered a relevant factor regulating seed dormancy processes. Several genetic approaches utilizing mostly mutant and transgenic lines of *Arabidopsis thaliana* and Solanaceae species have established that ABA is involved in induction and maintenance of dormancy, whereas GAs release dormancy and promote germination [12]. Other hormones including ethylene, brassinosteroids, auxin and cytokinins have been also proposed to affect dormancy and germination. The molecular factors and pathways conditioning seed dormancy status have been enumerated in several recent reviews [13]–[16]. Early studies showed that the orthologous B3 class transcription factors encoded by *VIVIPAROUS 1* (*VP1*) in maize and *ABA-INSENSITIVE 3* (*ABI3*) in *A. thaliana* are involved in seed development and dormancy [17], [18]. The basic leucine zipper (bZIP) transcription factor encoded by *ABA-INSENSITIVE 5* (*ABI5*) interacts with ABI3 and mediates its effect on the expression of ABA responding genes thought the ABA-response element ABRE [19], [20].

Bud dormancy in perennial plants resembles seed dormancy at the hormonal level [21]. The involvement of ABA in bud dormancy events is suggested by multiple physiological and transcriptomic studies [22]–[26], though few genetic approaches support this statement [27], [28]. The DORMANCY ASSOCIATED MADS-box (DAM) group of transcription factors related to SHORT VEGETATIVE PHASE (SVP) of *A. thaliana*, have been proposed to regulate bud dormancy processes in peach [29], leafy spurge [30] and Japanese apricot [31]. *DAM* gene expression correlates with the dormancy state of buds, with higher transcript accumulation during the cold season followed by chilling-dependent down-regulation prior to dormancy release [30], [32], [33]. Different transcriptomic approaches have been conducted in order to identify *DAM*-like and other genes related to dormancy at the expression level [27], [34]–[41].

In order to find common molecular features between seed and bud dormancy processes, we investigated the expression of *DAM* and other bud dormancy-associated genes, involved in transcriptional regulation, ABA response and metabolism, and desiccation tolerance, during the stratification of seeds in peach. The elucidation of general regulation pathways in both seed and bud structures may contribute to improve our basic knowledge on dormancy mechanisms, and be employed in plant breeding projects that profit from an early prediction of chilling requirements for blooming of new genotypes.

## Results

### Effect of Stratification on Seed Germination and Seedling Development in Peach

**Figure 1:**
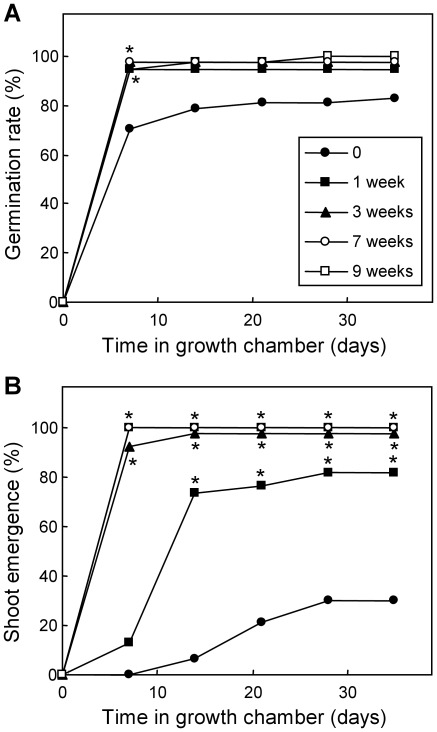
Effect of chilling on seed embryo germination and shoot emergence. The germination rate (A) and percentage of shoot emergence (B) were measured at different times after discrete periods of stratification: one week (black squares), three weeks (black triangles), seven weeks (white circles), nine weeks (white squares) and no stratification (black circles). Samples having non-overlapping binomial confidence intervals (95%) with the untreated sample are labelled with an asterisk.

An *in vitro* culture experiment was performed in order to characterize the response of peach embryos to different periods of cold stratification. The early variables germination rate, defined as the rate of embryos showing an at least 2-mm long radicle, and shoot emergence were measured after 0, 1, 3, 7 and 9 weeks of chilling treatment. After few days, the germination rate was nearly identical and total in embryos with periods of cold stratification of one week or longer, whereas non-stratified embryos showed a lower rate of about 80% (Figure 1A). The stratification also improved shoot growth. Embryos with three weeks and longer periods of chilling showed a significantly higher percentage of shoot emergence than one-week and non-stratified samples from the first growth measurements, whereas shoot emergence in embryos stratified for one week was higher than in untreated samples at 14 days and longer growing times (Figure 1B).

**Figure 2:**
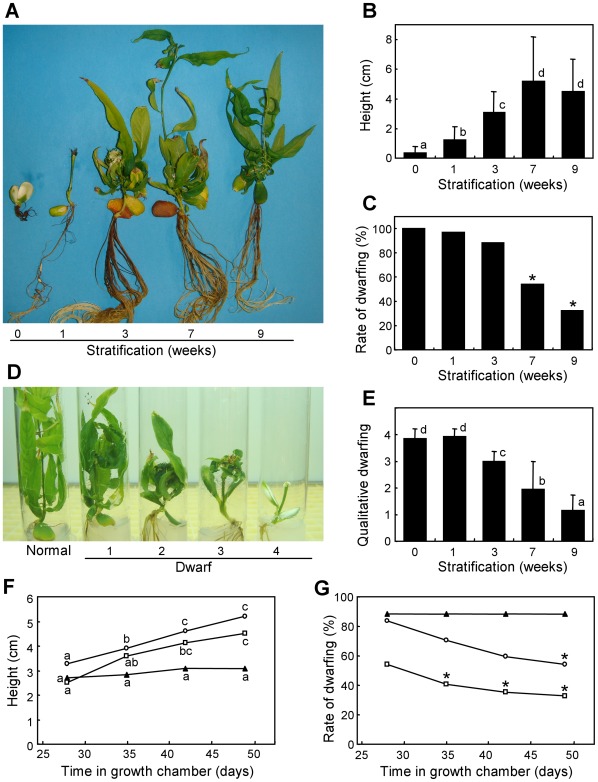
Effect of chilling on the physiological dwarfing of seedlings. (A) Representative plants subject to different stratification periods were photographed at the end of the *in vitro* experiment. (B) Average height of seedlings after seven weeks in growth chamber. (C) Percentage of dwarfed plants after seven weeks in growth chamber. (D) Representative seedlings showing different qualitative levels of dwarfism: level 1 individuals were slightly shorter than normal ones and had both normal and curved leaves; level 2 seedlings had shorter internodes and mostly abnormal leaves; level 3 dwarfism led to small deformed leaves grouped in a rosette-like structure; and finally level 4 dwarfs had a drastic reduction of growth and barely recognizable leaves. (E) Average qualitative dwarfing of seedlings grown for seven weeks. The height (F) and rate of dwarfing (G) of seedlings obtained from embryos stratified for three (black triangles), seven (white circles) and nine weeks (white squares), were measured at different times. In (B) and (E), error bars represent standard deviation, n>35. Non-overlapping letters (a-d) indicate significant difference within (F) or between different stratification treatments (B,E), based on ANOVA analysis and Multiple Range Tests procedure with a confidence level of 95%. Asterisks indicate non-overlapping binomial confidence intervals (95%) with non-stratified samples (C) or samples stratified for 3 weeks (G).

In addition to these early observations on germination and shoot emergence, germinated embryos showed long-term effects of chilling on seedling development. At the end of the *in vitro* experiment, seedlings with 0–1 weeks of stratification were small and not viable in most cases, whereas those with 3–9 weeks of chilling were larger with variable rates of physiological abnormalities, such as dwarfing (Figure 2A). The height of seedlings grown *in vitro* was significantly improved by the cold treatment, with optimal values in plants stratified for 7 and 9 weeks (Figure 2B). The rate of dwarfed individuals was also lower after 7 and 9 weeks of stratification (Figure 2C). A qualitative classification of physiological dwarfs attending to the height of the plant, the presence of rosettes and the size and form of leaves was performed, which served to assign dwarfing values from 1 (those dwarfs more similar to normal plants) to 4 (those with deeper symptoms) (Figure 2D). According to this classification, we found that chilling reduced the qualitative dwarfing level in a similar way to the dwarfing rate (Figure 2E). Plants stratified for 7 and 9 weeks grew actively during a time interval of three weeks, while those stratified for 3 weeks hardly increased their average height during the same period (Figure 2F). Interestingly, plants stratified for 7 and 9 weeks also reduced their rate of dwarfing in this time interval, due to an overall recovery of growth by the appearance of lateral shoots with normal development (Figure 2G).

### Regulation of Gene Expression in Stratified Seeds

**Figure 3:**
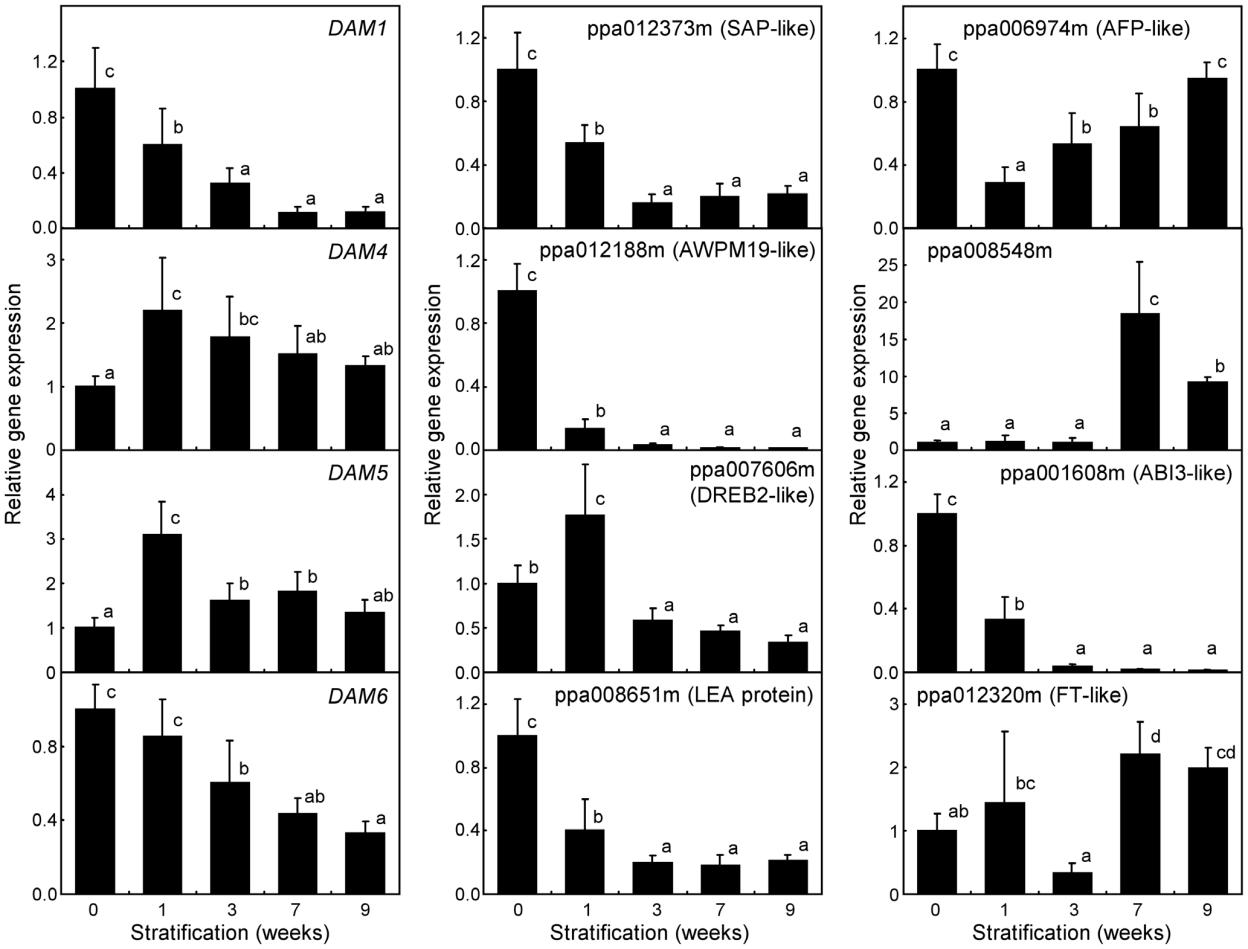
Expression of selected genes in stratified embryos. Relative expression of bud-dormancy related genes was determined by quantitative real-time RT-PCR with specific primers (Table S1). The name of the gene or transcript model is shown in the upper side of the graph. Expression levels are relative to Actin. An expression value of one is assigned to the non-stratified sample. Data are means from two biological samples with 2–3 technical replicates each, with error bars representing standard deviation. Non-overlapping letters (a–d) indicate significant difference between different stratification treatments, based on ANOVA analysis and Multiple Range Tests procedure with a confidence level of 95%.

Previous transcriptomic approaches performed in our group, based on transcript enrichment by suppression subtractive hybridization (SSH) and cDNA microarray hybridization, led to a set of genes differentially expressed during bud dormancy release in peach [26], [41]. We selected several of these genes, previously validated by quantitative real-time RT-PCR on buds, for its expression analysis in peach embryos subject to the stratification treatments described above. The genes *DAM1*, *DAM4*, *DAM5* and *DAM6* are components of the *DAM* multigene family coding for MADS-box transcription factors involved in bud dormancy regulation in peach and other species. The expression of *DAM* genes was fairly detectable by quantitative real-time RT-PCR in peach seeds, in contrast with the lack of detectable *DAM* expression by northern analysis reported in leafy spurge seeds [30]. *DAM1* and *DAM6* reduced gradually and significantly their expression during the chilling treatment to reach their lowest values in 7 and 9 weeks samples (Figure 3). However, *DAM4* and *DAM5* transcripts were accumulated during the first week under stratification and subsequently reduced at longer time samples.

We assayed other genes down-regulated during bud dormancy release, related to elements of the ABA and drought stress response in *A. thaliana* [26]. The genes coding for STRESS ASSOCIATED PROTEIN (SAP)-like (peach transcript model ppa012373m), AWPM19-like (ppa012188m), DEHYDRATION-RESPONSIVE ELEMENT BINDING PROTEIN2 (DREB2)-like (ppa007606m), and LATE EMBRYOGENESIS ABUNDANT (LEA) protein (ppa008651m) reduced drastically their expression in stratified embryos (Figure 3), as previously observed in peach buds subject to environmental chilling. The *ABA-INSENSITIVE FIVE BINDING PROTEIN* (*AFP*)-like gene showed an early down-regulation in the first chilling week, followed by a slow recovery to reach initial expression values. Most of genes investigated with a higher expression in buds after dormancy release had a negligible expression in embryos, but ppa008548m coding for a putative cinnamoyl-CoA reductase showed detectable expression after 7 and 9 weeks of stratification.

We analyzed by quantitative real-time RT-PCR two additional transcripts that were not previously identified in our transcriptomic experiments, but were considered putative candidates to affect dormancy responses in seeds and buds. The *ABA-INSENSITIVE 3* (*ABI3*)-like transcript (ppa001608m) strongly declined during the first three weeks of chilling, whereas *FLOWERING LOCUS T* (*FT*)-like (ppa012320m) had higher expression values in last stratification stages (Figure 3).

### ABA Content Rapidly Decreases During Stratification

**Figure 4:**
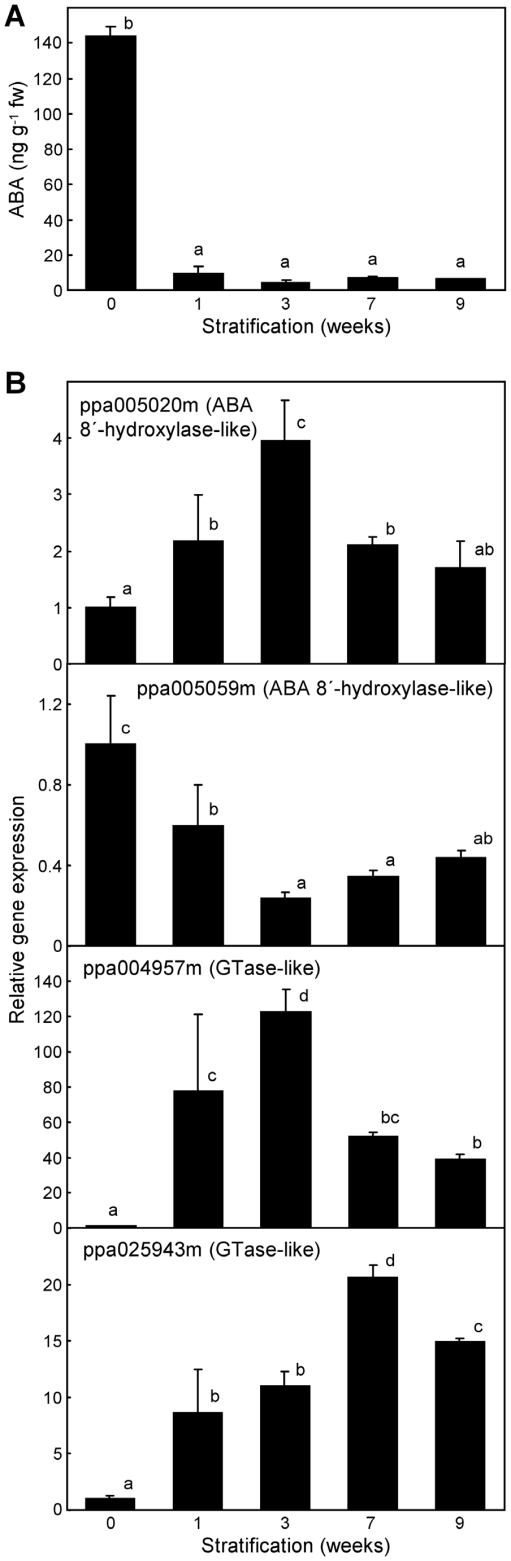
Abscisic acid (ABA) content and expression of ABA metabolic genes in stratified embryos. (A) ABA content in the embryo was determined as detailed in Materials and Methods. (B) Relative gene expression by quantitative real-time RT-PCR of peach genes coding for putative ABA degrading enzymes, as ABA 8′-hydroxylase and ABA glucosyltransferase (GTase), based on protein similarity to known enzymes in other species. Expression levels are relative to Actin. Data are means from three biological replicates (A) or two biological samples with 2–3 technical replicates each (B), with error bars representing standard deviation. The significant difference between treatments, using ANOVA and Multiple Range Tests with a confidence level of 95%, is indicated by different letters (a-d).

The ABA content in stratified embryos decreased from about 140 ng per gram (fresh weight) to less than 10 ng after one week of chilling. The hormone content did not change significantly after longer chilling treatments (Figure 4A). No genes have been described in peach related to ABA degradation, but protein similarity searches led to the identification of ppa005020m and ppa005059m, highly similar to ABA 8′-hydroxylase genes *CYP707A2* from *A. thaliana* and *CYP707A1* from *Prunus avium* respectively [42], [43]; and ppa004957m and ppa025943m, similar to ABA glucosyltransferase gene *UGT71B6* from *A. thaliana* [44]. An expression analysis of these genes revealed that ppa005020m, ppa004957m and ppa025943m were significantly up-regulated during one-week stratification (Figure 4B), and thus one or more of them could account for the observed decrease of ABA in the embryo.

**Figure 5:**
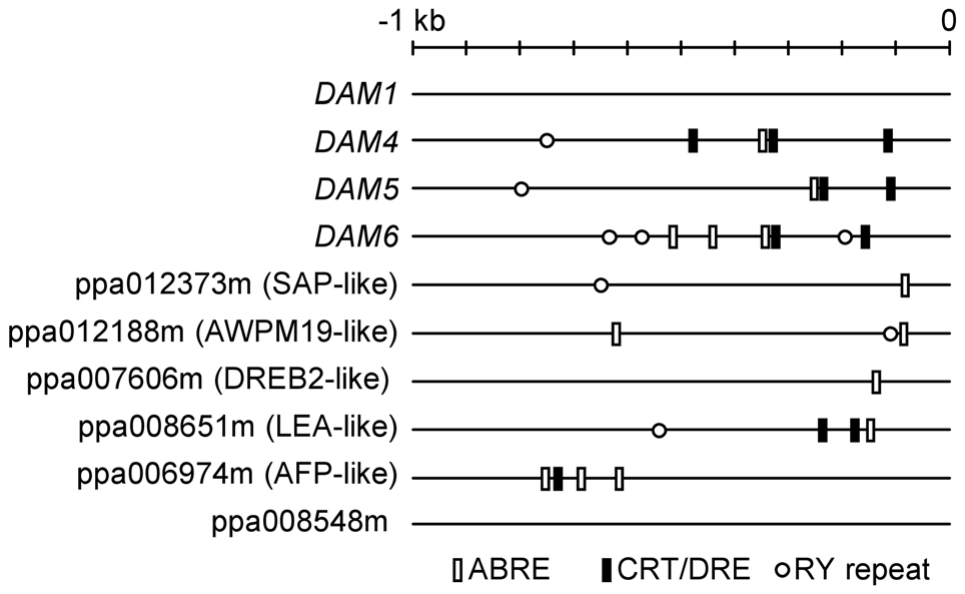
Predicted cis-elements in the promoter of chilling-regulated genes. The ABA-response element (ABRE, white rectangles), C-repeat/dehydration-responsive element (CRT/DRE, black rectangles), and seed-specific RY repeats (white circles) were localized in the 1-kb upstream sequence from the transcriptional start site when known, and from the translation start (in ppa006974m and ppa008548m), of chilling affected genes, as described in Materials and Methods.

In order to identify cis-regulatory elements responding to ABA and drought stress in the genes analyzed by quantitative real-time RT-PCR, we examined 1 kb sequence of their promoters beginning from the transcription start when known, otherwise the translation start ATG was used. We performed a search of ABA-responsive elements (ABRE), C-repeat/dehydration-responsive elements (CRT/DRE), and RY repeats as described in Materials and Methods. Genes repressed by chilling treatment had at least one of these three elements in their promoters with the exception of *DAM1* gene, and four of them had the three elements (Figure 5). We did not find any of these regulatory sequences in the promoter of the ppa008548m gene induced by cold stratification.

### Phenylpropanoid Metabolites Accumulate During Prolonged Cold Treatment

**Figure 6:**
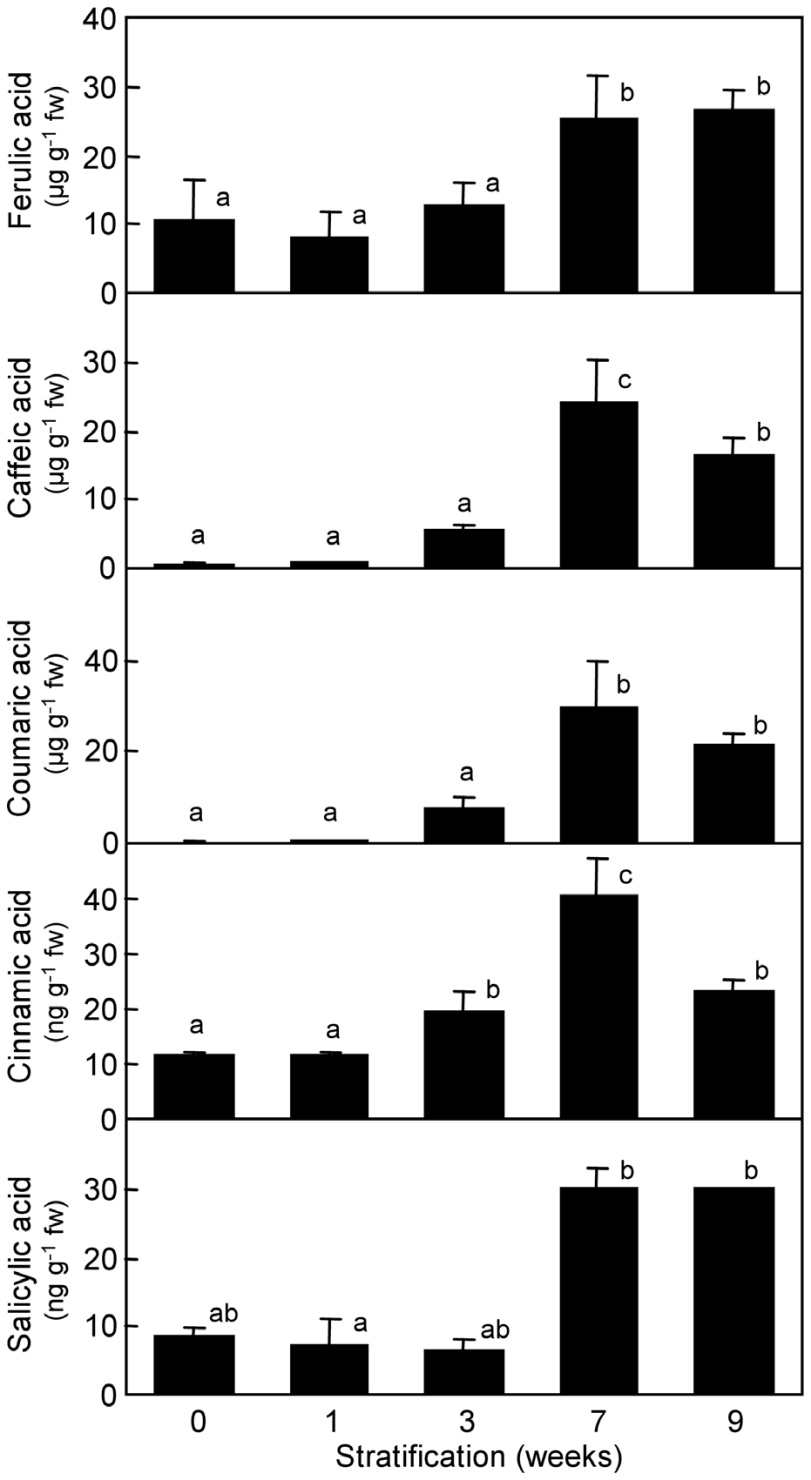
Accumulation of phenylpropanoids in stratified embryos. Ferulic, caffeic, coumaric and cinnamic acids, and the phytohormone salicylic acid were determined in embryos after different chilling treatments. Data are means from three biological replicates, with error bars representing standard deviation. Different letters (a-c) indicate significant difference between treatments, based on ANOVA and Multiple Range Tests with a confidence level of 95%.

A systematic analysis of metabolites was performed in stratified embryos. We found several intermediate metabolites of the phenylpropanoid biosynthesis pathway accumulating in embryos stratified for 7 and 9 weeks. Phenylpropanoids are compounds synthesized from the amino acid phenylalanine that lead to the lignin precursors monolignols through the activity of cinnamoyl CoA reductase and other enzymes. Interestingly ppa008548m gene, coding for a cinnamoyl CoA reductase-like, was also found expressed in 7 and 9 weeks samples after quantitative real-time RT-PCR analysis (Figure 3), which suggested a link between both data. Among these phenylpropanoids, the ferulic acid approximately doubled its initial content during long chilling treatments (Figure 6). Over-accumulation of other compounds from this pathway was even more pronounced. Caffeic, coumaric and cinnamic acids increased respectively 40-fold, 100-fold and 4-fold after a stratification period of 7 weeks, followed by a slight decrease two weeks later. Interestingly, the hormone salicylic acid increased its content during the chilling treatment in a similar way to phenylpropanoid metabolites (Figure 6). However, the addition of salicylic acid to the culture medium at different concentrations did not ameliorate the germination or physiological dwarfing defects observed in peach seeds (data not shown).

## Discussion

### Stratification has Short and Long-term Effects on Peach Germination and Development

Short chilling treatments of one and three weeks were sufficient for optimal germination and shoot emergence, respectively. These chilling requirements for radicle and shoot growth were lower than those reported by other physiological studies [45], [46], which may be due to genotype-based differences or most likely to previous removal of the seed coat in our samples. Coat excision helped to discard the mechanical and physiological contribution of the coat to seed dormancy, which in consequence was exclusively dependent on the embryo component. This experimental procedure was essential to distinguish the observed effects of cold stratification on both, seed germination and seedling dwarfing. Thus, in addition to early benefits of chilling on germination and shoot emergence, longer chilling treatments of seven and nine weeks reduced the physiological dwarfing of seedlings. This double effect of stratification on germination and subsequent development of seedlings has been noted previously in stone-fruit species [8], [47], but no molecular mechanisms have been proposed to explain it. The improved germination ratio observed after one week of chilling was associated to a drastic reduction in ABA content, in close agreement with the known role of this hormone in the induction and maintenance of dormancy in seeds. However, the alleviation of dwarfing at long chilling treatments was not related to changes in ABA, which precludes a function of ABA in this seedling developmental abnormality. The peach genes ppa005020m, ppa004957m and ppa025943m, coding respectively for an ABA 8′-hydroxylase-like and two ABA glucosyltransferase-like genes were induced by stratification, which suggests that one or more of them could be involved in ABA removal and consequently modulate embryo dormancy.

Other investigated compounds, as salicylic acid and the phenylpropanoids ferulic, caffeic, coumaric and cinnamic acids, followed a modification pattern opposite to ABA. They accumulated in embryos stratified for seven to nine weeks, coinciding with a lower rate of seedling dwarfing. Overproduction of these compounds could benefit the normal development of seedlings or simply be a biochemical feature of dormancy-released embryos prepared to develop normally, however we have not obtained experimental evidences supporting any of these hypotheses. The phenylpropanoids pathway results in the synthesis of the monolignols p-coumaryl, coniferyl and sinapyl alcohols, which are the main precursors of lignin polymers, through the sequential activity of cinnamoyl CoA reductase (CCR) and cinnamyl alcohol dehydrogenase (CAD) enzymes [48]. Lignin deposition contributes to the secondary thickening of the cell wall, and takes part in xylem cell differentiation. The synthesis of this complex polymer becomes detectable in early stages of seedling development in *A. thaliana*, associated to the developing vasculature [49]. Interestingly, a triple *A. thaliana* null mutant in one CCR and two CAD genes, leading to a strong reduction in lignin deposition, displayed a severe dwarf phenotype and abnormal leaf morphology [50], resembling the symptoms of physiological dwarfing described in this work. These observations and the high-chilling dependent induction of ppa008548m in stratified embryos (Figure 3), coding for a predicted protein similar to CCR, suggest the participation of phenylpropanoids and lignin biosynthesis pathways in the alleviation of anomalies associated to physiological dwarfing.

### Bud and Seed Dormancy Regulate a Common Set of Genes

The quantitative real-time RT-PCR analysis of genes previously related to bud dormancy revealed a parallel pattern of gene expression in buds and embryos. Several genes down-regulated during bud dormancy release after the fulfilment of cultivar-specific chilling requirements were also repressed by cold stratification in embryos. This suggests the presence of common regulatory pathways in dormancy release mechanisms of buds and seeds, although it might also be due to the effect of common environmental factors acting on the expression of certain genes without any regulatory role in dormancy release. The *in silico* search of known homologous genes in other species and cis-elements in their promoters pointed to a common effect of ABA and drought signalling on such genes. However ABA could only account for transcript accumulation changes observed during the first week of chilling, due to the stabilization of ABA content after this time. Consequently, later down-regulation of the expression of these genes should be assigned to additional regulatory pathways. Drought and cold share common regulatory mechanisms through the CRT/DRE elements; thus imbibition and chilling of seeds caused by the stratification treatment could exert contradictory effects on the expression of genes showing this element. The intensity and the time response of drought and cold signals could be different and consequently affect differently to gene expression values measured in this work.

*DAM* genes are particularly interesting among this set of genes. *DAM* have been related to bud dormancy maintenance by expression and functional studies in multiple species, and are considered the major known regulators of this process [26], [29], [30]–[33], [38]. The fact that *DAM1*, *DAM5* and *DAM6* are also significantly repressed during chilling treatment of the embryo suggests their participation in mechanisms of transcriptional regulation associated to release of seed dormancy by stratification, but additional experimental evidences are required to confirm it. Recently, *DAM5* and *DAM6* genes have been found modified by long genomic insertions into their first intron in the peach ‘Okinawa’, a low chilling rootstock showing lower *DAM5* and *DAM6* expression in buds [51]. Interestingly, ‘Okinawa’ seeds also had a positive germination response to stratification periods at 7°C [52].

Previous molecular studies relating seed germination and flowering in *A. thaliana* have been recently published. The *FLOWERING LOCUS C* (*FLC*) gene coding for a MADS box transcription factor involved in flowering time regulation through the vernalization pathway, also affected the temperature-dependent germination of dormant seeds [53]. The effect of *FLC* on seed germination was most likely mediated by *FLOWERING LOCUS T* (*FT*), which has also been shown to play a role in meristem growth and possibly dormancy per se [54]–[56], and which appears to be regulated by *DAM* genes [30], [31]. Interestingly, a peach gene similar to *FT* increased its expression after stratification during 7–9 weeks (Figure 3), which points to the presence of a related signalling pathway in peach. The RNA Polymerase II Associated Factor 1 Complex (PAF1C) of *A. thaliana* has been also proposed to have a dual role in flowering and seed dormancy [57]. Moreover, a poplar orthologue of *ABSCISIC ACID INSENSITIVE 3* (*ABI3*) gene, involved in the ABA-dependent expression of many seed-specific genes in *A. thaliana*, is expressed in buds during bud set and causes some alterations in bud development when overexpressed and silenced [22].

The transcriptional similarities between bud and seed dormancy highlighted in this work may also be relevant for plant breeding purposes. The selection of early and late flowering genotypes from a segregating population usually requires the arduous evaluation of large collections of individuals, which could be improved by a previous selection of the desirable trait at the seed level. Previous studies found a positive correlation between the chilling requirements for seed germination and blooming in almond and apple [58], [59]. This work contributes to characterize the molecular bases underlying these and other physiological observations with high interest to plant breeders.

## Materials and Methods

### Plant Material and *in vitro* Culture

The *Prunus persica* L. Batsch cv ‘Big Top’ was grown in an orchard located at the Instituto Valenciano de Investigaciones Agrarias (IVIA) in Moncada (Spain) under standard agricultural practices. Mature fruits were collected and immediately broken to eliminate the endocarp with special scissors. Seeds were disinfected and flamed with alcohol, and then the coats were removed under sterile conditions. Embryos were cultured in a sterile Woody Plant Medium [60], solidified with 0,8% Bacteriological Agar and distributed in 20 ml aliquots into 25×150 mm culture tubes. Chilling treatment or stratification was performed by storing the tubes at 4°C in continuous darkness for 0, 1, 3, 7 or 9 weeks. After the stratification period, 8–10 embryos were frozen with liquid nitrogen and stored at –80°C for RNA extraction and RT-PCR, and 48 embryos (tubes) were placed in a culture chamber at 24°C. The embryos were maintained in darkness during the first week and then in 16 hours light-photoperiod conditions for the rest of the development. The germination and shoot emergence rates, height of seedlings, and dwarfing phenotype were noted once per week.

### Quantitative Real-time RT-PCR

Total RNA was isolated from 100 mg of seeds deprived of their coats using the RNeasy Plant Mini Kit (Qiagen, Valencia, CA, USA), but adding 1% (w:v) polyvinylpyrrolidone (PVP-40) to the extraction buffer before use. From 8 to 10 individuals were pooled for each treatment. We performed two RNA extractions per sample, which were treated independently until cDNA synthesis, constituting two biological replicates. One microgram of total RNA was reverse transcribed with SuperScript III First-Strand Synthesis System for RT-PCR (Invitrogen, Carlsbad, CA, USA) in a total volume of 20 µl. Two microliter of a 40X diluted first-strand cDNA were used for PCR reactions in a final volume of 20 µl. Quantitative real-time PCR was performed on a StepOnePlus Real-Time PCR System (Life Technologies, Carlsbad, CA, USA), using Perfecta SYBR Green SuperMix ROX (Quanta Biosciences, Gaithersburg, MD, USA). Primer pairs are listed in Table S1. Cycling protocol consisted of 10 min at 95°C, followed by 40 cycles of 15 s at 95°C for denaturation, and 1 min at 60°C for annealing and extension. Specificity of the PCR reaction was assessed by the presence of a single peak in the dissociation curve after the amplification and through size estimation of the amplified product by agarose electrophoresis. We used as reference a peach Actin transcript amplified with specific primers. Relative expression was measured by the relative standard curve procedure. Results were the average of two independent biological replicates with 2–3 technical replicates each.

### Plant Hormone and Metabolite Analyses

Plant hormones and phenolics were analyzed by LC/ESI-MS-MS essentially as described [61]. Briefly, fresh frozen plant material was extracted in ultrapure water using a tissue homogenizer (Ultra-Turrax, Ika-Werke, Staufen, Germany) after addition of 50 µl of a mixture of internal standards (see [61] for further details). After extraction and centrifugation, pH of the supernatant was adjusted to 3.0 and partitioned twice against di-ethyl-ether (Panreac, Barcelona, Spain). The organic layers were combined and evaporated in a centrifuge vacuum evaporator (Jouan, Saint-Herblain, France). The dry residue was thereafter resuspended in a water:methanol (9∶1) solution, filtered and injected in a HPLC system (Alliance 2695, Waters Corp., Milford, MA, USA). Analytes were then separated in reversed-phase Kromasil 100 C18 column (100×2.1 mm, 5 µm particle size, Scharlab, Barcelona, Spain) using methanol and 0.01% acetic acid in water as solvents at a flow rate of 300 µl min^–1^. The mass spectrometer, a triple quadrupole (Quattro LC, Micromass Ltd., Manchester, UK) was operated in negative ionization electrospray mode using N_2_ as nebulization and desolvation gas and set at 100 and 800 l h^-1^, respectively. During measurements, capillary voltage was set at 3.5 kV whereas cone voltage was adjusted for every analyte. The precursor and product ions as well as cone and collision voltages were selected after direct injection of pure commercial standards into the mass spectrometer.

### Statistical Analysis

Significant changes of height, level of qualitative dwarfing, metabolite content and gene expression between different stratification treatments or growth times were evaluated by one-way analysis of variance (ANOVA) using the STATGRAPHICS Centurion XVI software (StatPoint Technologies, Warrenton, VA, USA). Multiple Range Tests were performed in order to determine which sample means were significantly different from the others, using the Least Significant Difference (LSD) method of Fisher with a confidence level of 95%. Significant differences were labelled with different letters from “a” to “d” in graphic representations.

The variables germination rate, percentage of shoot emergence and rate of dwarfing were compared between stratification treatments by confidence intervals in the case of binomial, as proposed by Clopper and Pearson [62]. Stratified and untreated samples were considered significantly different when their 95% confidence intervals were not overlapping, shown by an asterisk label in the graph.

### Promoter Analysis

The promoter sequence of transcript models was obtained from peach genome database at phytozome (http://www.phytozome.net/cgi-bin/gbrowse/peach/). We selected 1-kb upstream sequence from the transcriptional start site when known, and from the translation start ATG in ppa006974m and ppa008548m genes. The ABRE element was located in these sequences applying a matrix-based procedure [63]. The core sequence of the CRT/DRE element (G/A)(C/T)CGAC was screened manually [64]. Finally, the RY-repeat element involved in seed-specific expression was searched using the Plant Cis-acting Regulatory DNA Elements Database (PLACE) [65].

## Supporting Information

Table S1 Primers employed in the quantitative real-time RT-PCR.
(XLS)
